# Everybody's Page

**Published:** 1890-02-15

**Authors:** 


### Everybody's Page.

The doctors of the Belgian expedition are introducing vaccination among the natives of the Lower Congo.

An English doctor is reported to have cured 30 cases of facial neuralgia and headache by causing his patients to snuff powdered salt up their nose. The remedy is simple.

M. Fougue, the mineralogist, by a combination of silicate of copper and lime has obtained the beautiful azure colour found in the ruins at Pompeii. It is said to be perfectly unchangeable.

A digestible way of cooking fish for a delicate person is to put a small white fish like sole into a jelly-jar with a tablespoonful of milk and a little parsley. Cover very closely with paper and set the jar in a saucepan of boiling water at the side of the fire for half-an-hour.

Clot Bey, the founder of modern medicine in Egypt, says that it requires as much surgery to kill one Egyptian as seven Europeans, and there is no doubt that Egyptians bear surgical operations with extraordinary pluck and success. A man in a native hospital who has had his thigh amputated at two o'clock is sitting up and quite lively at six.

The cost of cremating bodies at Père Lachaise is very different now to what it was at first. In the new crematorium, which was opened in August last, they use coke instead of wood, and the outlay has diminished from thirty-five to three francs. Formerly it took an hour and three-quarters to reduce a body to ashes, and now it is accomplished in one hour and a-quarter.

Everything goes by express train in these days—work, pleasure, everything; it can't be helped, we must go on with the tide; but unless we are very careful our health is likely to suffer. Perhaps people who are absolutely drones, who do nothing, who have, or who think they have, no responsibilities in this world, can let their life run on in a "come what will" sort of manner. Those who are the busy bees of the world, who rush on early and late in incessant action, must take care that they do not hurry themselves [to an untimely end, by letting their brains be in a continuous state of morbid activity. Leisure is essential. How are we to get it, somebody will say; and we answer, it may be very difficult, but have you ever tried method? A little planning out of our days and life, and especially doing everything in the proper time—it is wonderful how it answers. Method is generally abused by people who have never tried it.

In a short article in the *Medical Record* Dr. Percy Crandall tells us that the natives of all classes in Hayti believe that phthisis is both contagious and infectious, and a person who is suffering from the disease is avoided by everybody, and should he die the whole of his property is destroyed, and sometimes houses are burned right down for fear of infection. Dr. Crandall, riding once in Northern Hayti, came upon a sort of marsh, which was filled with household goods of all descriptions, pianos, sewing machines, &c., also the bleached bones of animals. This was the "Cémetière des Chevaux," wherein were thrown the bodies of dead animals, and any furniture or belongings which were found in the room in which a consumptive had died. No native would touch a single thing once thrown in the cemetery. Phthisis affects women in Hayti more than men, and their sedentary habits, the damp earthen floors of their houses, and the high temperature are probably some of the causes of the frequency of the disease. No Haytian will ever admit he is in consumption.

Electrical execution is to be investigated by the Medical section of the French Academy of Science.

The Berlin President of Police has issued a report warning people that the American evaporated apples have been found to contain salts of zinc.

At Nordhausen, in Saxony, an order has been issued to barbers that they are to disinfect their brushes, &c., after each persons use; an excellent order if carried out, which we may be permitted to think a trifle doubtful.

Dr. Ord states he has obtained good results in "thrush" from the use of an application of black-wash and glycerine in equal parts, applied by a small camel-hair brush. The quantity used is so small as to be quite harmless.

A temperance mission is evidently wanted at Johannesburg. It appears from a statement recently made that the total expenditure in alcoholic drinks for three months was £260,800, or at the rate of £1,043,000 a-year, being £61 per head of the adult population per annum. If this is correct the drink bill comes to two-thirds of the gross value of the gold produced at the mines!

A theatrical performance by the deaf and dumb sounds paradoxical, but the performance last week of "Richard III." by children of the asylum in Old Kent Road, shows to what perfection instruction in the "oral" system has been brought. The audience was much impressed by the clearness of the articulation of the children, who spoke their parts quite intelligibly, and as though they understood fully the meaning of what they were saying.

Some people worry over every little obstacle which upsets the evenness of their lives; others irritate us by their seeming indifference to ups and downs; but, on the whole, we are inclined to think that the latter class have the best of it. There is no doubt that a fretting person is a nuisance to himself and everybody else. Naturally, some are physically stronger than others, and it is easier for them to overcome difficulties; but the weaker members of society might educate their feelings to a more philosophical tendency if they would but try. Worrying increases with indulgence at a terrific rate, and who does not know how much more satisfaction we derive when we are in trouble by going to a calm, self-possessed person instead of to another of the worrying description, who, not content with struggling with present difficulties, begins to look about at once for more "breakers ahead."

The *Grantham Journal* had a delightful story *àpropos* of the influenza epidemic. We fancy—but we may be mistaken—that somehow or other we have heard a very similar little story, but, then, history repeats itself. A flagman on a much-frequented line of railway believed that he, like everybody else, was attacked by influenza, so he applied to the company for advice from their medical man. The doctor, called here and there by the influenza'd public, could not easily find time to go to the far-off village where the man lived, so he wrote to him suggesting he should stand at a certain point where a certain train would pass in which the doctor was travelling, and put his tongue out, and thus the doctor would be able to see what was the matter. The flagman obeyed—simple-minded countryman!—but he caused a slight disturbance by his obedience. It is really rather a funny picture, the infuriated British public who had travelled by the train writing to the company to complain of the insolence of one of their officials.

